# Measuring Health Behaviors in Populations

**Published:** 2010-06-15

**Authors:** Ali H. Mokdad, Patrick Remington

**Affiliations:** Institute for Health Metrics and Evaluation, University of Washington; University of Wisconsin School of Medicine and Public Health, Madison, Wisconsin

## Abstract

Health behaviors are a leading cause of illness and death in the United States. Efforts to improve public health require information on the prevalence of health behaviors in populations — not only to target programs to areas of most need but also to evaluate the effectiveness of intervention efforts. Telephone surveys, such as the Centers for Disease Control and Prevention's Behavioral Risk Factor Surveillance System, are a good way to assess health behaviors in populations. These data provide estimates at the national and state level but often require multiple years of data to provide reliable estimates at the local level. With changes in telephone use (eg, rapid decline in the ownership of landlines), innovative methods to collect data on health behaviors, such as in health care settings or through Internet-based surveys, need to be developed.

## Introduction

Efforts to improve community health at the national, state, and local level require detailed and accurate information about the prevalence of health behaviors (1-3). If existing data collection systems are to remain viable, current approaches to measuring population health behaviors must be adapted. Potential solutions address the challenges of nonresponse, coverage, data quality, sample size, and costs.

McGinnis and Foege summarized the role of health behaviors as a leading cause of death and labeled them the "actual causes of death" (4). Later updated by Mokdad (5), these studies concluded that approximately half of all deaths in the United States could be attributed to factors such as smoking, physical inactivity, poor diet, and alcohol use (Table 1). Public health campaigns were established that educated the public about the need for healthy lifestyles and supported health-promoting programs and policies. These changes contributed to major declines in heart disease, stroke, and injury deaths (6).

Telephone surveys emerged as a feasible method to assess the prevalence of many health risk behaviors among populations (7). In 1984, the Centers for Disease Control and Prevention (CDC) implemented the first state-based surveillance system for health behaviors, the Behavioral Risk Factor Surveillance System (BRFSS) (8). BRFSS collects information on health risk behaviors associated with the leading causes of illness and death (9).

## Reasons for Measuring Health Behaviors

The measurement of health behaviors in populations is useful for both program planning and program evaluation. For program planning, estimates of the prevalence of behavioral risk factors can be used to set priorities or to compare rates across communities. For example, to provide more reliable estimates, the Wisconsin County Health Rankings combines 7 years of data from BRFSS to compare the rates of behaviors across all the counties in the state (10). In contrast, more precise measures are needed when evaluating changes in health behaviors over time. For example, a 95% confidence limit of plus or minus 3% may be sufficient to estimate the prevalence of smoking in a population but is insufficient to demonstrate changes in smoking rates over time. Efforts to reward communities for improved health outcomes (11) would require precise estimates of health behaviors so that incentives could be closely linked with the implementation of programs or policies.

## Methods to Measure Health Behaviors in Populations

Several methods exist to assess behaviors in a target population. The choice of methods is usually a function of cost due to time and personnel. Ideally, a census would be the optimal means of collecting data. However, censuses are not conducted frequently enough to enable timely data for planning. Hence, surveys are often the best mode of data collection. Advances in sampling techniques and software availability have rendered surveys the workhorse for behavioral assessment. Several modes are useful for collecting survey data: 1) face to face, 2) telephone, 3) mail, or 4) Web. The mode dictates whether the data are self-reported, observed, or measured.

Five components determine the quality of a survey: 1) coverage, 2) sampling, 3) nonresponse, 4) measurements, and 5) data processing. Adequate coverage is achieved when the sampling frame includes all units of the population of interest. If the list of population units is incomplete, frame coverage errors result. Challenges to coverage vary by survey mode. Usually, sampling frames for face-to-face surveys are expensive to develop, whereas telephone sampling frames are challenging because of the use of cellular phones and number portability (area codes are no longer associated with a specific geographic location). The US Postal Service's sampling frames (mail surveys) are not complete, but they are improving. On the other hand, Web sampling frames are not yet comprehensive.

Adequate sampling is achieved when each element on the sampling frame has a known and nonzero probability of selection. This protects against sampling bias and enables the researchers to quantify sampling error. Again, this error varies by survey modes. Face-to-face and telephone surveys have well-developed techniques for sampling. On the other hand, mail surveys do not have a clear method for within-household selection, although some promising findings have been reported. Researchers cannot control who will answer the questionnaire once the letter is received.

Nonresponse errors occur when researchers are unable to obtain data from selected respondents. This error has 2 aspects. Unit nonresponse means that the selected person refuses to do the survey; item nonresponse means that the respondent completes the survey but refuses to answer certain questions. Again, this error varies by survey mode and questions. For example, in face-to-face interviews, a respondent may be less likely to provide personal information on sexual behaviors to an interviewer. However, the same person may provide such answers via the Web or through a computer-assisted interview (ie, researchers provide respondents a laptop during the household interview, allowing them to self-administer sensitive questions).

Measurement errors occur when a respondent's answer to a question is inaccurate (departs from the "true" value). Several factors contribute to this error, primarily, the wording of questions and their order in the questionnaire. Therefore, it is crucial to cognitively test questionnaires and pilot surveys before full implementation. Survey mode has implications for measurement errors (interviewer vs self-administered). Indeed, the interviewer stimuli and the manner in which the survey questions are conveyed to respondents and responses are recorded will affect this error. For example, asking "Are you trying to lose weight?" or "Weight loss is important for your health; are you trying to lose weight?" will yield different estimates for weight-loss attempts.

Data processing errors occur during data management, editing, and recoding. Sometimes errors are made during imputations of certain missing items or responses. Finally, errors could be made in the calculation of final weights or poststratification adjustments. Hence, systems must be in place during survey operation for quality assurance and control.

## Existing Surveys of Health Behaviors

Several US surveillance systems and surveys provide valuable information on behavioral risk factors (Table 2). Most of the surveys and surveillance systems are national; a few exceptions provide data at the local and state levels. In addition, most of the surveys use self-reported information on health behaviors because of the high cost of face-to-face surveys and collecting physical measurements. Among self-reported surveys, telephone surveys are the most common because they are the least expensive. In addition, the development of computer-assisted telephone interviewing software has allowed for a rapid release of data.

The largest telephone survey in the United States is BRFSS, whereas the National Health Nutrition Examination Survey (NHANES) is the main survey to provide physical measurement. A brief description of some of the key surveys follows.

### The Behavioral Risk Factor Surveillance System

BRFSS is a state-based system of health surveys (9,12). The objective of BRFSS is to collect uniform, state-specific data on health risk behaviors, clinical preventive health practices, and health care access that are associated with the leading causes of death and illness in the United States. Currently, data are collected monthly in all 50 states, the District of Columbia, Puerto Rico, the Virgin Islands, and Guam. Health departments use the data to identify demographic variations in health-related behaviors, target services, address emergent and critical health issues, propose legislation for health initiatives, measure progress toward state and national health objectives, and design evaluations of their programs and policies. For most states and counties, BRFSS is the only source of population-based health behavior data related to chronic disease.

### National Health and Nutrition Examination Survey

NHANES is a series of national surveys of American health and nutrition that have been conducted since the early 1960s (13). The surveys obtain both interview and physical examination data from national samples of the US population. Data collection for the current NHANES began in 1999 and is ongoing. Each year, nearly 7,000 people of all ages in households across the United States are randomly selected to participate. The study design includes representative samples of people by age, sex, and income and oversamples African Americans, Mexican Americans, adolescents, older people, and pregnant women. Participants are interviewed in their homes. After the interview is complete, they are asked to participate in a series of physical examinations. Physical exams are conducted in specially equipped and designed Mobile Examination Centers consisting of 4 trailers. NHANES data have been widely used by policy makers at the national level.

### Pregnancy Risk Assessment Monitoring System

The Pregnancy Risk Assessment Monitoring System (PRAMS) is a surveillance project of CDC and state health departments (14). PRAMS collects state-specific, population-based data on maternal attitudes and experiences before, during, and shortly after pregnancy. Research has indicated that maternal behaviors during pregnancy may influence infant birth weight and mortality. The goal of the PRAMS project is to improve the health of mothers and infants by reducing adverse outcomes such as low birth weight, infant illness and death, and maternal illness. PRAMS provides state-specific data for planning and assessing health programs and for describing maternal experiences that may contribute to maternal and infant health.

### The Youth Risk Behavior Surveillance System

The Youth Risk Behavior Surveillance System (YRBSS) monitors priority health-risk behaviors and the prevalence of obesity and asthma among youth and young adults (15). YRBSS includes a national school-based survey conducted by CDC and state, territorial, tribal, and local surveys conducted by state, territorial, and local education and health agencies and tribal governments. YRBSS monitors 6 categories of priority health-risk behaviors among youths and young adults, including behaviors that contribute to unintentional injuries and violence; tobacco use, alcohol and other drug use; sexual behaviors; and diet and physical inactivity.

### The National Survey on Drug Use and Health

The National Survey on Drug Use and Health (NSDUH) provides yearly national and state-level data on the use of alcohol, tobacco, and illicit and nonmedical prescription drugs in the United States (16). Other health-related questions also appear from year to year, including questions about mental health. Many state health agencies use NSDUH data to estimate the need for drug treatment facilities.

### Other surveys and surveillance systems

Among other surveys and surveillance systems that states can use for their public health activities are the Pediatric Nutrition Surveillance System, Pregnancy Surveillance System, and the National Health Care Surveys (Table 2).

## Examples of Data Use at the State and Local Level

### Trends in obesity by state

BRFSS provides valuable information about health behaviors at the state and local level that is of interest not only to public health professionals but also to the media. The use of a standard questionnaire in all states and over time enables researchers to compare the health of communities. The best known example of using data to communicate information about the obesity epidemic is in a landmark article in 1999, followed by the posting of PowerPoint slides on the CDC Web site (http://www.cdc.gov/obesity/data/trends.html). These slides graphically show the spread of high rates of obesity across the entire United States, from coast to coast (17-19).

### The SMART Project

The need for prevalence estimates at the local level has led to the creation of the Selected Metropolitan/Micropolitan Area Risk Trends (SMART) Project to analyze the data of selected metropolitan and micropolitan statistical areas (MMSAs) that have 500 or more respondents in BRFSS. Although BRFSS was designed to produce state-level estimates, growth in the sample size has facilitated production of smaller-area estimates. SMART showed that the prevalence of certain behaviors varied across cities, not unlike the differences found across states. Researchers were able to observe variation in prevalence by comparing cities with their surrounding metropolitan areas and with the rest of their state. This new use of BRFSS data fills a public health need for local area surveillance data to support targeted program implementation and evaluation; these data should help cities to better plan and direct their prevention efforts.

### Mandating colorectal cancer screening insurance coverage

Data show that screening for colorectal cancer lags far behind screening for other cancers. In 2006, BRFSS data showed that New Mexico's colorectal cancer screening rates were below the national median. Citing BRFSS data, which indicated that states with mandatory coverage had better colorectal cancer screening rates, New Mexico's legislature passed a law requiring health insurance providers to cover colorectal cancer screening for New Mexico residents aged 50 years or older, joining 22 other states with mandatory coverage.

## Discussion

Data from surveys of health behaviors in populations will continue to play a role in public health efforts at the national, state, and local level. During the past 30 years, telephone surveys have become a standard approach to collect information from adults and children. However, as response rates continue to decline and costs to increase, other methods for collecting these data need to be considered.

### Challenges of health behavior surveys and data

The challenge for surveys and surveillance systems is to effectively manage increasingly complex systems that can serve the needs of multiple programs while adapting to changes in communications technology such as the increased use of cellular telephones and call screening devices, societal behaviors (concerns about privacy and declining participation in surveys), and population diversity (the growing number of languages spoken in the United States, more cultural and ethnic diversity). As a result, all surveys are facing declining response rates, especially those based on telephones. Hence, all surveys are focusing on efforts to improve their data quality, reach populations previously not included in their survey, and expand the usefulness of the surveillance data.

Many surveys have established expert panels to guide their system improvements, to ensure the quality and validity of the data, and to reduce the potential for bias in estimates. In addition, surveillance is becoming more expensive and funding is becoming a major challenge. Indeed, many behavioral surveillance surveys are receiving less funding at a time of more demand to increase their sample sizes and add more questions.

Many surveys and surveillance systems face these challenges and are exploring potential solutions (Appendix). Some provide incentives to increase response rates. In addition, most large surveys are using prenotification to increase participation in their systems. Multimode data collection can also increase coverage and reduce cost. The systems maximize the collection of data using a less expensive mode (eg, Web or landline telephones) and contacting fewer respondents from more expensive modes, such as household interviews. The combination would allow a more representative sample of the community at a lower cost.

Moreover, different participants may prefer certain modes and will respond better to such options. For example, young participants may prefer to respond to a survey over the Internet and may be more accessible through their cellular telephones. To address self-reported bias, surveys could consider conducting physical measurements on a subsample of their respondents to examine and adjust for this limitation. All surveys and surveillance systems should institute a transparent data-quality report for their users to better describe the limitation of the data and its generalizability. Finally, all surveys should consider rotating questions every year or every several years; fewer questions make better use of the questionnaire's limited space and reduces the burden on respondents.

### Future directions for health behavior surveys and surveillance systems

Several issues should be considered in moving forward with data collection and local needs. The survey and surveillance community should develop and implement more innovative methods for data collection that will reduce operational cost, hence allowing for an increase in sample size. The key factor is how much detailed information is needed for monitoring trends and for action. Unless the risk factor is very rare or prevalent only in a subgroup of the population (eg, the percentage of people diagnosed with diabetes receiving a yearly eye exam), a survey based on a sample size of 300 or more should be adequate for action. On the other hand, monitoring a trend is more challenging, especially if the purpose is to detect a small change in the prevalence of a risk factor. In reality, the changes that we would expect in behaviors after a program or policy change are very small. In such a case, researchers would need a larger sample size to detect a significant difference from a baseline.

Several approaches are available for acquiring data for local communities. The preference would be to increase the sample size of an ongoing survey in a community. However, such an option can be very expensive. Perhaps using the existing infrastructure of health care settings to collect data is worth pursuing. This approach would involve developing new statistical methods to combine data from different sources to inform decision makers. The use of small-area estimates is the most promising alternative. Indeed, using existing methods and a small sample size, it is possible to provide valid estimates at the local level.

Showing the values of surveillance systems at the local level is the best way to secure resources. Moreover, it is time to critically review our surveillance systems to explore the possibility of combining efforts and systems to better meet the needs of local data. For example, the National Immunization Survey could be combined with BRFSS and NHANES could be combined with the National Health Interview Survey (ie, measurements on a subsample of NHIS). Indeed, CDC is now better positioned to implement such changes to improve surveillance, having recently created the Office of Surveillance, Epidemiology, and Laboratory Services. The future of health behavior surveys and surveillance systems depends on such improvements to ensure adequate funding for data collection, more research on alternative methods for data collection, and ongoing support for the use of these data.

## Figures and Tables

**Table 1:** Actual Causes of Death, United States, 1990 and 2000

**Actual Cause**	**N (%)** a ** in 1990**	**N (%)** a ** in 2000**
Tobacco	400,000 (19)	435,000 (18)
Poor diet and physical inactivity	300,000 (14)	365,000 (15)
Alcohol consumption	100,000 (5)	85,000 (4)
Microbial agents	90,000 (4)	75,000 (3)
Toxic agents	60,000 (3)	55,000 (2)
Motor vehicle	25,000 (1)	43,000 (2)
Firearms	35,000 (2)	29,000 (1)
Sexual behavior	30,000 (1)	20,000 (<1)
Illicit drug use	20,000 (<1)	17,000 (<1)
Total	1,060,000 (50)	1,124,000 (47)

a The percentages are for all deaths. Source: reference 5.

**Table 2:** Major US Surveys That Measure Health Behaviors

**Acronym**	**Name**	**Sponsoring Agency**
ACS	American Community Survey	US Census Bureau
BRFSS	Behavioral Risk Factor Surveillance System	CDC
CPS	Current Population Survey	US Census Bureau
CSFII	Continuing Survey of Food Intakes by Individuals	US Department of Agriculture
CSHCN	National Survey of Children with Special Health Care Needs	CDC
IFPS	Infant Feeding Practice Study II	CDC
IHIS	Integrated Health Interview Series	NCHS-NHIS
HRS	Institute for Social Research Health and Retirement Study	University of Michigan, Institute for Social Research
LSOAs	Longitudinal Studies of Aging	CDC-NCHS
MEPS	Medical Expenditure Panel Survey	AHRQ
NAMCS	National Ambulatory Medical Care Survey	CDC
NAS	National Asthma Survey	CDC
NCS	National Children's Study	NIH
NCS-1	National Comorbidity Survey Replication	ICPSR
NEHIS	National Employer Health Insurance Survey	CDC
NHAMCS	National Hospital Ambulatory Medical Care Survey	CDC
NHANES	National Health and Nutrition Examination Survey	CDC
NHCS	National Health Care Surveys	CDC
NHDS	National Hospital Discharge Survey	CDC
NHHCS	National Home and Hospice Care Survey	CDC
NHIS	National Health Interview Survey	CDC
NIS	National Immunization Survey	CDC
NLAAS	National Latino and Asian American Study	ICPSR
NLTCS	National Long Term Care Survey	Duke University
NMFS	National Mortality Followback Survey	CDC
NMIHS	National Maternal and Infant Health Survey	CDC
NNHS	National Nursing Home Survey	CDC
NOES	National Occupational Exposure Survey	CDC
NSAS	National Survey of Ambulatory Surgery	CDC
NSCH	National Survey of Children's Health	CDC
NSDUH	National Survey on Drug Use and Health	SAMHSA, US Census Bureau
NSECH	National Survey of Early Childhood Health	CDC
NSFG	National Survey of Family Growth	CDC
PedNSS	Pediatric Nutrition Surveillance System	CDC
PNSS	Pregnancy Surveillance System	CDC
YRBSS	Youth Risk Behavior Surveillance System	CDC

Abbreviations: CDC, Centers for Disease Control and Prevention; NCHS, National Center for Health Statistics; NHIS, National Health Interview Survey; AHRQ, Agency for Healthcare Research and Quality; NIH, National Institutes of Health; ICPSR, Inter-University Consortium for Political and Social Research; SAMHSA, Substance Abuse and Mental Health Services Administration.

## Appendix. Selected Challenges and Potential Solutions for Surveys of Health Behaviors

**Table:**

**Challenge**	**Potential Solution**
Nonresponse	Consider incentives and prenotification.
Coverage due to mode of data collection	Consider multimode collection.
Self-reported data	Consider measurements on an in-person subsample.
Coverage (young age groups and language barriers)	Consider use of cellular phones and employ bilingual interviewers.
Data quality	Institute data quality protocols and checks.
Small sample size	Oversample in certain areas, use small-area techniques.
Cost	Charge for certain questions, use survey for evaluation and get funding as part of the intervention, use multimode data collection.
Limited space for questions	Consider rotating surveys.
